# Plasmodesmata-Related Structural and Functional Proteins: The Long Sought-After Secrets of a Cytoplasmic Channel in Plant Cell Walls

**DOI:** 10.3390/ijms20122946

**Published:** 2019-06-17

**Authors:** Xiao Han, Li-Jun Huang, Dan Feng, Wenhan Jiang, Wenzhuo Miu, Ning Li

**Affiliations:** 1 College of Biological Science and Engineering, Fuzhou University, Fuzhou 350116, China; hanxiao@caas.cn; 2 College of Forestry, Central South University of Forestry and Technology, Changsha 410004, China; nghua@126.com (L.-J.H.); jiangwenhan1999@126.com (W.J.); alic5e@163.com (W.M.); 3 Biotechnology Research Institute, Chinese Academy of Agricultural Science, Beijing 100081, China; gygzgzyx@126.com; 4 State Key Laboratory of Cultivation and Protection for Non-Wood Forest Trees, Ministry of Education, Central South University of Forestry and Technology, Changsha 410004, China

**Keywords:** plant cell walls, plasmodesmata, callose, callose synthase

## Abstract

Plant cells are separated by cellulose cell walls that impede direct cell-to-cell contact. In order to facilitate intercellular communication, plant cells develop unique cell-wall-spanning structures termed plasmodesmata (PD). PD are membranous channels that link the cytoplasm, plasma membranes, and endoplasmic reticulum of adjacent cells to provide cytoplasmic and membrane continuity for molecular trafficking. PD play important roles for the development and physiology of all plants. The structure and function of PD in the plant cell walls are highly dynamic and tightly regulated. Despite their importance, plasmodesmata are among the few plant cell organelles that remain poorly understood. The molecular properties of PD seem largely elusive or speculative. In this review, we firstly describe the general PD structure and its protein composition. We then discuss the recent progress in identification and characterization of PD-associated plant cell-wall proteins that regulate PD function, with particular emphasis on callose metabolizing and binding proteins, and protein kinases targeted to and around PD.

## 1. Introduction

Plasmodesmata (PD) span the plant cell walls and connect protoplasts of adjacent cells [1,2]. The presence of PD allows molecular communication conveniently between plant cells [3,4,5]. Several thousand PD form a network between fully differentiated plant cells [6], transforming individual cells into interconnected symplasts, providing channels for the intercellular exchange of electrical signaling, diffusion of small molecules, and transportation of macromolecules such as proteins, nucleic acids, and viruses [7,8,9,10]. The exchange channel provides a direct cell-to-cell cytoplasmic pathway for plant molecule transport and information transmission [11]. The PD also regulates plant metabolic activity and gene expression through the transfer of signaling chemicals, enabling plant cells to exchange information with each other to coordinate physiological processes [12,13,14].

Although being a functional important structure, the molecular composition of PD remains largely elusive [15,16]. PD are embedded in rigid cell walls; it is arduous to purify and study such an organelle via classical biochemical methods. High-resolution electron microscopy showed that proteinaceous particles are associated with PD structures [17]. Studies such as immunolocalization revealed that various proteins are present in PD, including kinase-like proteins [18], actin, and myosin [19], which may serve as permanent structural components. In addition, genetic and biochemical approaches applied to dissect PD-associated functional components led to the discovery of callose binding proteins and receptor-like kinase proteins in the model plants [20]. They regulate the permeability of PD and control intercellular substance transport and signal communication. PD-mediated symplasmic transport is involved in a variety of plant physiological processes that are pivotal for the development of plants of economic interest, such as shoot apex dormancy, flowering, fruit ripening, fiber elongation, and plant–microbe symbiosis [21]. Understanding PD function through characterization of PD-related proteins may have the potential for improving agronomic traits in the future.

It is generally believed that callose deposited at the PD neck region plays a regulatory role in the neck switch [22]. This is the most well-known mechanism for regulating PD permeability [23]. Callose is a polysaccharide in the form of β-1,3-glucan [24]. Callose is synthesized at the plasma membrane at the neck region of PD, squeezing the plasma membrane inwardly, thereby narrowing down the neck region, which reduces the free space that PD can use for molecular passage [25]. A large amount of evidence indicates that the change in PD permeability is due to the synthesis and hydrolysis of callose in the cell wall [26,27,28]. Callose synthase, β-1,3-glucanase, and PD-associated callose binding protein are involved in the regulation of callose homeostasis in PD, which in turn affects the permeability of PD channels [29,30].

## 2. The Formation and Structure of PD

From the perspective of origin, PD is generally formed during the mitotic cell division. In the primary formation of PD, although the protoplasm is split by the newly formed cell plate, the endoplasmic reticulum (ER) is still connected through the cell plate [31]. The presence of the ER in the channels prevents the deposition of wall-forming substances, thus maintaining the connection between the two separated cells [32]. Furthermore, the ER transforms into the PD under the pressure of the cell plate or membrane. There are also PD between non-sister cells, that is, PD can be formed between non-mitotic cells [6,33]. However, the molecular origin and process of secondary PD formation are not well understood.

Ding et al. [34] obtained a series of new PD structural details using rapid freezing and freezing replacement sample preparation techniques, high-resolution electron microscopy, and computer-aided image processing techniques. A structural model of PD was proposed based on their observations. At both ends of PD is an enlarged hole [35,36], and the center of the PD is a cylindrical body formed by the appressed ER, called a desmotubule [37]. The desmotubule is tightly constricted with little space [38]. Cytoskeletal proteins are often found in PD, such as actin and myosin [39,40]. Protein particles of about 3 nm are embedded between the appressed ER and the plasma membrane. Electron-dense radial fibrils connect the protein particles [34,41]. The protein particles on the ER membrane present a spiral or a series of circularly arranged rotations. In the cross-section, 7–9 particles can be seen [34]. The channel of the ER is considered to be the transport pathway of PD [42,43]. The two ends of the channel become smaller, called the neck region [37].

Actin filaments and callosum sphincters are two modes of controlling PD permeability. According to experimental observations, actin filaments are located near and inside the PD channel, and the polymerization state of actin changes the permeability of PD [44,45]. On the other hand, callose aggregates at the neck region narrow the space between cell wall and PD desmotubule. 

## 3. PD-Associated Structural Proteins

As mentioned above, studies such as immunolocalization revealed that various proteins such as actin, myosin, tubulin, and calreticulin are present in PD as structural components [39,46]. Actin and myosin are components of the cell dynamic network, cytoskeleton, involved in intracellular transport [47]. Microtubule arrays consist of tubulin heterodimers which play critical roles in cell division and intracellular organization [48]. These proteins are not cell-wall proteins, but are associated with the plant cell-wall structure of PD in the context of this review (Table 1).

### 3.1. Actin

Actin exists in the entire PD [60]. The systematic observation of the nature and structure of PD in the epidermal cells of *Allium sativum* L. was performed using a fluorescence probe and confocal microscope combined with transmission electron microscopy and immunogold labeling [49]. White et al. [49] indirectly demonstrated the presence of actin in PD in young and mature cell walls of *Hordeum vulgare* and *Nicotiana plumbaginifolia* by colloidal gold labeling. The results effectively confirmed the presence of F-actin in PD. It is unclear how the filaments are organized within PD. They may exist in the lumen between the desmotubule and the plasma membrane, connecting the cytoskeleton between adjacent cells [45,61]. The actin filaments may also function as routes along which vesicular trafficking is possible. Treatments with actin disrupter, cytochalasin D, and actin-stabilizing fungal toxin, phalloidin, showed opposite effects on the cell-to-cell transport of a fluorescent reporter dextran; the latter chemical severely restrained the dextran movement [49,50]. These results indicate that the PD structural component may also be involved in regulating the PD permeability, with larger molecules being able to move through once actin filaments are disrupted.

### 3.2. Myosin

Immunochemical localization studies indicated that myosin was also an integral component of PD [49,51,52]. Radford and White’s experiments indirectly demonstrated a myosin distribution on PD [52]. They used an animal myosin antibody with colloidal gold to prepare an immuno-electron microscopic sample of onions, maize, and *Arabidopsis* seedlings. They found colloidal gold-labeled particles on the cytoplasm and PD, suggesting that myosin might be present in PD. To date, myosin is confirmed to belong to 15 families, of which only some are found in plants, where the myosin found in PD belongs to the eighth family. The effects of myosin inhibitor treatments somehow led to inconsistent results as to the role of myosin in PD [39]. How myosin regulates PD function is still under debate.

### 3.3. Tubulin

Blackman and Overall systemically characterized whether the cytoskeletal-related proteins were targeted to PD and found tubulin in the extracts of the PD-containing internode cell walls of corals, whereas it was not detected in the PD-independent internode cell walls, suggesting that PD may contain tubulin [51]. The role of tubulin in PD is less well characterized as compared to actin and myosin [62]. Blackman and Overall [51] speculated that tubulin may be indirectly involved in long-distance transport.

## 4. PD-Associated Regulatory Proteins

Callose is deposited at the cell wall near the neck of the PD and regulates the PD size exclusion limit (SEL) [22]. The PD function in plants is closely linked to callose deposition in the PD neck region. PD-associated regulatory proteins are more or less callose-related (Table 1). As mentioned above, the level of callose in PD is controlled by two antagonistic callose metabolic enzymes, callose synthase (CALS), also well known as glucan synthase-like (GSL), and β-1,3-glucanase (BG), and requires other enzymes that regulate callose stability [24,35]. In addition, the PD permeability changes involved in plant development and defense, processes involving callose, also require other proteins associated with callose [63,64].

### 4.1. Callose Synthases Modify Cell Wall PD to Regulate Plant Development

In the callose-dependent PD permeability, the PD channel is turned off by a high level of callose and turned on by a low level of callose, indicating that the callose level of the PD neck region is particularly crucial to PD function. The callose deposition determines PD SEL, which in turn determines their permeability and the transportation of macromolecules. Biochemical and genetic studies in barley and tobacco pollen tubes first demonstrated that GSLs produced callose [65]. This is also evidenced by studies in *Arabidopsis* [36,66]. GSLs control developmental signals by modulating the amount of callose in PD.

Plant hormones play a role in many aspects of plant physiology and development. Auxin has a gradient distribution in plants and plays an important role in plant growth and development [67]. As a small molecule, auxin can diffuse freely through the PD [68]. A recent study revealed that the auxin gradients can be maintained by controlling the PD permeability. Han et al. [53] screened all 12 *GSL* gene mutants in *Arabidopsis*, and found that the PD callose content decreased significantly only in the *gsl8/cals10* mutant. They used hypocotyls that responded to the auxin gradient distribution as experimental systems and found that the inducible *gsl8* RNA interference (RNAi) system showed lower phototropism and agravitropism in hypocotyl, which mimics auxin defects. Further studies found that a decrease in the messenger RNA (mRNA) level of *GSL8* resulted in the decrease of callose at PD, the increase of permeability of PD, and the increase of the diffusion of auxin transported by the symplast; finally, the gradient distribution of auxin was destroyed. Moreover, they proposed that the auxin gradient in plant tissues was established by the auxin–GSL8 feedback circuit associated with auxin response factor 7 (ARF7).

PD-mediated signaling plays a role in plant morphogenesis. Callose biosynthesis in the regulation of symplasmic transport by PD plays a central role in plant cell-to-cell signaling, but it is not clear how this regulation is arranged [69]. Vatén et al. [36] screened for the *gsl12/cals3* mutant in an ethyl methanesulfonate (EMS) mutagenesis library of *Arabidopsis*. Observation of the GFP::GSL12 fusion protein expressed in the leaves indicated that it was localized in PD. The gain-of-function mutation of *GSL12* leads to a transient accumulation of callose in PD in the early stage of root development, a decrease in PD SEL, a decrease in intercellular transport, and a defect in root development. This suggests that the regulatory activity of GSL12 influences the biosynthesis of callose in the cell wall surrounding the PD, which determines the pore size of the PD and regulates the transport of substances. The experiment with the *GSL12* allele (*cals3m*) showed that the PD pore size between plant tissues is controlled spatially and temporally. Using this system, they assessed the movement of the short-root (SHR) protein, microRNA165/6, and cytokinin in *Arabidopsis* roots and revealed the role of symplasmic communication in lateral root development. The above results indicate that GSL12 is a callose synthase, and that the biosynthesis of callose affects the plant developmental signals transmitted by PD. Interestingly, Yadav et al. [66] also identified GSL12 in *Arabidopsis*, and its semi-dominant mutants overproduce callose in PD. 

The new cells required for plant growth and development are derived from the differentiation of stem cells. There is a group of stem cells called stem-cell niche (SCN) at the root apical meristem, and at its center are slowly dividing cells called the quiescent center (QC). It was believed that the location information provided by intercellular communication enabled QC and SCN to be maintained, but it was not verified for a long time. Liu et al. [70] developed a system that can effectively block PD-mediated communication in QC. They controllably and specifically blocked the intercellular communication between QC and neighboring cells by expressing *icals3m* (inducible form of *cals3m*) in QC. Using this system, they explored the function of intercellular communication in the SCN. They found that changes in symplastic communication between QC and adjacent cells resulted in elevated local auxin concentrations and the establishment of AP2-domain transcription factor gradients. Changes in symplasmic communication not only affect the polar transport of auxin, but also affect the biosynthesis of local auxin, which together provide local information for stem cell maintenance and play a key role in the maintenance of SCN. These studies revealed a direct link between symplasmic translocation and auxin-dependent regulation of stem-cell identity.

Slewinski et al. [71] identified a callose synthase in maize. They obtained *tie-dyed* (*TDY*) mutants of maize exhibiting green and yellow variegated leaves by EMS mutagenesis, and the yellow leaf tissue accumulated too much soluble sugar and starch. After constructing the population, they cloned the *TDY2* gene using map-based cloning, and found that *TDY2* encodes a callose synthase. Characterization of expression patterns by RT-PCR analysis, RNA in situ hybridization, and colocalization with the vascular marker, PINFORMED1a (ZmPIN1a)-YFP, revealed that *TDY2* was preferentially expressed in vascular tissues of developing young leaves. The movement experiments of ^14^C-labeled sucrose and carboxyfluorescein diacetate (CFDA) showed that the *tdy2* leaves defected in the entry of sucrose into phloem. Solutes could not enter phloem in yellow leaf region of *tdy2* mutants, but its long-distance transport capacity was not impaired. The ultrastructure study of the *tdy2* mutant vein by transmission electron microscopy showed that the phloem and xylem cells in the yellow leaf region of *tdy2* were changed, and that the vein differentiation was incomplete, which hindered the symplasmic transport from phloem companion cells to sieve elements mediated by PD, resulting in functional defects in phloem. As to how the callose synthase TDY2 targets to PD in the cell walls, Baker et al. [72] hypothesized that TDY2 was synthesized in the ER and traveled through secretory vesicles from the Golgi apparatus to localize to PD, or that TDY2 proteins translocated through the secretory system to the plasma membrane and PD. 

Song et al. [73] identified a plasmodesmal callose synthase gene *cannot reach the roof 1* (*CRR1)* that affects ovary development in rice plants. The rice *CRR1* gene is closely related to *Arabidopsis GSL8* and *GSL10*. By aniline blue staining and immunocolloidal gold analysis, the *crr1* mutation had no effect on the callose deposition at root cell plates, pericarp, and pedicel sieve plates. However, iodine staining of ovarian development showed that the starch granules were significantly less numerous than wild type (WT) in the pericarp of the *crr1* ovary, and that the distribution was uneven, indicating that the unloading from the phloem to the pericarp could be defective. RT-PCR analysis and *pCRR1*::GUS reporter transgenic plants showed that *CRR1* was specifically expressed in vascular tissues. The movement analysis of CFDA revealed that sugars were inefficiently unloaded from the lateral vascular system to the developing caryopsis. Transmission electron microscopy showed that the connection between sieve tube cells and peripheral parenchyma cells of vascular bundles was reduced, and some cells had defective cell walls, where the deposition of callose at PD was reduced. These results suggest that CRR1 may alter the transport of cell fate determinants by controlling the permeability of PD, resulting in the change of the vascular cell differentiation pattern, thereby playing a key role in determining the plant reproductive development.

### 4.2. Callose Hydrolases Degrade Callose to Enhance Intercellular Communication

Class I β-1,3-glucanase (GLU I) plays an important role in the defense against virus infection [74]. Iglesias et al. [75] studied the role of GLU I in PD and PD-dependent communication. They obtained a homozygous class I β-1,3-glucanase (GLU I)-deficient mutant tobacco (TAG 4.4) by antisense transformation. This mutant become insensitive to viral infections. Observation was carried out of the size of the necrotic area and the GFP expressed by a recombinant form of potato virus X (PVX). GFP showed that the spreading of the virus in this mutant was slowed down. Observation of the fluorescence of CMV’s 3a movement protein in fusion with GFP (3aMP::GFP) revealed that the movement of viral movement proteins between cells was limited in TAG 4.4. Observations of fluorescein isothiocyanate (FITC)-dextrans and FITC-peptides indicated that plasmodesmal SEL was reduced in this mutant. Aniline blue staining showed that, after the loss of GLU I, the deposition of callose increased. The above results indicate that β-1,3-glucanase plays an important role in the metabolism of callose. It may promote the degradation of callose, change the permeability of PD, and regulate the movement of macromolecules through PD. Bucher et al. [76] further explored the relationship between GLU I and plant sensitivity to viruses. They studied the role of GLU I in viral migration between cells from both gain- and loss-of-function mutants. They inserted the different target fragments into the TMV-based expression vector to obtain products of different enzymatic activities. The positive insertion fragments expressed the active GLU I protein, and the necrotic area of leaves increased continuously after infection with the virus. The plants infected with viruses expressing antisense GLU I showed reciprocal effects. These experiments indicated that GLU I promoted the movement of virus between cells by hydrolyzing callose to increase PD SEL.

Levy et al. [28] identified a putative plasmodesmal associated protein β-1,3-glucanase (AtBG_ppap) in *Arabidopsis* and demonstrated that the permeability of PD is regulated by β-1,3-glucanase. The proteins in the PD-enriched fraction of *Arabidopsis* were separated by sodium dodecyl sulfate polyacrylamide gel electrophoresis (SDS-PAGE) and subjected to mass spectrometry analyses. The β-1,3-glucanase identified was named AtBG_ppap (beta-1,3-glucanase_putative PD-associated protein). Observation of transient and stable expression of GFP fusion proteins and aniline blue staining revealed that AtBG_ppap was related to the callose around PD in the cell walls. In the *Atbg_ppap* mutant, the callose level around the PD was higher, and the mobility of the GFP protein between cells was decreased, that is, the permeability of PD was lowered. The above experimental results demonstrated that AtBG_ppap is a PD-associated protein involved in plasmodesmal callose removal.

Intercellular communication regulates the function of individual cells to establish organ patterning and formation. It is long known that mobile signals are important in lateral root (LR) development. Paradoxically, the role of the intercellular channel PD in this process seems to have inadvertently not been elucidated. Recently, Benitez-Alfonso et al. [54] identified two plasmodesmal localized β-1,3-glucanase (PDBG) proteins that particularly accumulated in the LR founder tissue. Callose deposition changed correlating with changes in symplasmic transport during the course of LR development. During the formation of lateral meristems, callose amount was detected at low levels in PD. At early stages of LR development, callose accumulated to a moderate level between cells. Callose formation increased progressively when LRs developed. Such callose accumulation during LR organogenesis was regulated by two PDBGs, PDBG1 and PDBG2, which were localized in PD at early stages of lateral root primordia. In *pdbg1,2* double mutants, excessive callose deposition was detected around LR founder cells. In contrast, callose levels were significantly reduced in *PDBG1*-overexpressing plants. Consistent with callose accumulation levels, *pdbg1,2* double mutants showed a significant increase in LR formation. These results demonstrated that PD-dependent cell-wall glucanases play an important role in plant development via modulating intercellular communication. 

Furthermore, Rinne et al. [77] analyzed 10 presumed cell-wall β-1,3-glucanase genes belonging to the GH17 family (glucan hydrolase family 17) in poplar, and found that different treatments, such as photoperiod, chilling, and gibberellin, regulated these genes differently. Based on this, a model of breaking bud dormancy related to PD regulated by β-1,3-glucanase was proposed. Previous studies found that flowering locus T (FT) is the primary long-distance signal that is generated at the vascular bundle by activation of Constans (CO) and transported to the apex [78]. Centroradialis-like 1 (CENL1) in poplar is an ortholog of *Arabidopsis* TFL1 (terminal flower 1)—a hypothetical signal peptide, expressed in a small region under the shoot apical meristem (SAM) [79,80]. Both FT and CENL1 move through the phloem. They enter phloem tissue via PD which connect the cytoplasm of companion cells with the sieve tube cells. When the bud is dormant, SAM closes PD with callose, and cells in SAM become disconnected from each other [80]. Chilling induces FT and affects gibberellic acid 3 (GA3) biosynthesis by upregulating the expression level of *GA3-oxidase*; then, FT and β-1,3-glucanase proteins induced by GA3 reopen the PD in dormant buds. When the climate warms up, the reopened PD enables FT and CENL1 to move to their targets to cause buds to germinate. Following the study of the mechanism of seasonal dormant axillary buds (AXBs) releasing dormancy, Rinne et al. [81] continued to study the mechanism of semi-dormant axillary buds releasing dormancy during tree branch formation. AXB dormancy breaking also involved the GA pathway, and the expression of β-1,3-glucanase gene was induced by GA. They founded that the GA synthesis gene *gibberellin 3-oxidase 2* (*GA3ox2*) expression level in AXBs was very low; thus, GA was absent in AXBs. The expression of GA receptor gene *gibberellin insensitive dwarf 1-like* (*GID1-like*) gradually increased during the maturation of semi-dormant AXBs; thus, AXBs were sensitive to GA. They found that the expression of *GA3ox2* was significantly upregulated after decapitation of the hybrid poplar; thus, the biosynthesis of GA in AXBs increased. The GH17s have CBM43 modules that position them to callose around the PD. Upregulated GH17s responded to increased GA, degraded callose in the cell wall near PD, reducing callose deposition at PD, and promoted communication between stems and buds, thereby abolishing dormancy of semi-dormant AXBs. They studied the expression patterns of GA pathway and the *GH17s* gene at different stages of bud development, summarized their expression characteristics, and demonstrated the role of PD-related β-1,3-glucanases in bud development. Conversely, photoperiod regulates seasonal growth; prior to the advent of winter, short days (SDs) during late autumn promote bud dormancy to induce growth cessation. It was found that, in hybrid aspen trees, short photoperiods act through activation of the abscisic acid (ABA) pathway [82]. Later, gene expression analysis revealed that SVL (SVP-like), an ortholog of the *Arabidopsis* floral repressor short vegetative phase (SVP), was induced by SDs in an ABA-dependent manner. SVL negatively regulated the growth promotive GA pathways and positively mediated the expression of *callose synthase 1* (*CALS1*) in the shoot apex to induce callosic plugs in the neck region of PD [83,84]. Thus, PD-associated callose synthases and glucanases in the cell walls play important roles in plant development by regulating bud dormancy and floral transition.

Previous work showed that PD is momentarily closed during the elongation of cotton fibers in the allotetraploid species *Gossypium hirsutum* [85]. Ruan et al. [86] applied fluorescence probe carboxyfluorescein (CF), aniline blue staining, and immunoelectron microscopy to cotton genotypes with different cotton fiber lengths. It was found that the closure time of cotton fiber PD varied in different cotton genotypes, which was related to the deposition and degradation of callose at the base of cotton fiber. They cloned the *GhGluc1* gene from cotton fibers and found that it specifically encoded a β-1,3-glucanase localized to the fiber cells. Moreover, they also found that callose was degraded when GhGluc1 was expressed, and GhGluc1 expression was higher in plants with shorter cotton fibers. These results indicate that GhGluc1 is a callose degrading enzyme that allows the PD to be in an open state. Callose formation at the neck region closes PD. PD closure is positively correlated with fiber length; therefore, the callose catabolic enzyme GhGluc1 plays a negative role in fiber elongation.

### 4.3. PD-Associated Callose Binding Proteins (PDCBs) Regulate Callose Stability

Simpson et al. [29] identified a novel PD-related protein gene, *AT5G61130*, which encodes PD callose binding protein 1 (PDCB1) with callose binding activity in *Arabidopsis*. PDCB1 fused to the YFP coding sequence driven by the CaMV *35S* or its native promoter was stably transferred into *Arabidopsis* and transiently expressed in tobacco protoplasts. The fluorescent spots were found in the epidermal cell wall of *Arabidopsis* leaf tissue. Moreover, in spongy mesophyll cells, the fluorescent points were limited to the wall-to-wall junction between adjacent cells and still retained this pattern after plasmolysis. The results indicate that PDCB1 is associated with cell walls. Immunolocalization with anti-PDCB1 antiserum indicated that PDCB1 is located at the outer neck region of the PD. Combined with aniline blue staining and confocal microscopy, they proved that PDCB1 was colocalized with callose located in PD. They also studied PDCB2 (*AT5G08000*) and PDCB3 (*AT1G18650*) belonging to this family. A homology search of the *Arabidopsis* genome revealed that PDCB2 and PDCB3 have more than 50% amino-acid sequence similarity to PDCB1. Phylogenetic analysis revealed in fact that PDCB1, -2, and -3 lack a GPI-anchor signal sequence, but contain an X8 domain adjacent to the β-1,3-glucanase catalytic domain. This is in contrast to the previously identified PD-related β-1,3-glucanase [28]. By comparing with the structure of OLE-E9, the X8 domain-containing protein with callose binding activity in olives, it was found that the PDCB1 X8 domain is very similar to the OLE-E9 domain. Gel retardation assays showed that OLE-E10, PDCB1, and PDCB2 have callose binding activity in vitro. GFP diffusion assay showed that the migration of GFP decreased with the increase of PDCB1 expression, suggesting that the deposition of callose mediated by PDCB affected the symplastic communication through PD between cells.

PDCBs contain a carbohydrate binding module family 43 (CBM43) domain (also known as an X8 domain), as well as a callose binding domain, which can stabilize callose and regulate the dynamics of callose of PD [29]. PDCB1-overexpressing plants exhibited increased callose deposition in PD and decreased intercellular movement of reporter proteins. PDCBs are also predicted to be glycosylphosphatidylinositol anchored proteins (GPI-APS). Zavaliev et al. [87] studied the effects of GPI modification on PD proteins. They selected two GPI-APs associated with the callose of PD, BG_ppap and PDCB1 in *Arabidopsis*. They also chose two GPI-APs that were not associated with PD as control, arabinogalactan protein 4 (AGP4) and lipid transfer protein 1 (LTPG1). They first verified the subcellular localization of these proteins with monomeric citrine (mCitrine) labeling and then verified the modification of GPI with mannosamine. Subsequently, they subcellular localized the full-length sequence and deleted GPI signal sequence, respectively. It was found that PD targeting of BG_ppap and PDCB1 was mediated by GPI, and that the GPI modification played a major role in protein targeting to PD; furthermore, this sorting happened before positioning.

### 4.4. PD-Located Receptor-Like Proteins (PDLPs) Regulate Plant Defense Responses

PDLPs are PD-localized transmembrane proteins. PDLPs consist of a short cytosolic tail and two extracellular cysteine-rich receptor-like kinase domains of unknown function (DUF26) [88,89]. In *Arabidopsis*, eight PDLPs were identified by homologous comparison [56]; PDLP1, PDLP5, and PDLP6, were later found in independent proteomic screens of PD-enriched membrane [55,57]. PDLP1 (*AT5G43980*) is the first member of this protein family identified by cell-wall proteomics in *Arabidopsis* [55]. PDLP1 belongs to the DUF26 domain family. Thomas et al. [58] developed *Arabidopsis* transgenic plants overexpressing *PDLP1* and performed GFP diffusion experiments, confirming that PDLP1 can regulate intercellular transport by regulating PD. Moreover, they found through deletion analysis that their single transmembrane domain (TMD) is required for PDLP1a targeting to PD.

When fungi and oomycetes enter host plants with a haustoria structure, they cause plant defense responses, and the haustoria structure is gradually entrapped by the callose produced by the host. Caillaud et al. [59] studied the molecular mechanism of accumulation of callose around haustoria. They infected *pPDLP1*::GUS and *pPDLP5*::GUS reporter transgenic plants with pathogens, and found that *PDLP1* was specifically expressed in cells containing haustoria and responded to pathogen infection. Observation of PDLP1::GFP showed that PDLP1 localized at PD in both uninfected and infected tissue, and PDLP1 was associated with haustoria at the early stages of pathogen invasion in infected tissue. Moreover, the signaling peptide that targets PDLP to haustoria is located at its C-terminus, allowing it to be localized to the haustorial membrane. Significantly more callose was detected in transgenic plants overexpressing *PDLP1* than in non-transgenic plants following pathogen infection. The experimental results of Caillaud et al. [59] directly demonstrate that PDLP1 contributes to the immune defense against pathogens by affecting the deposition of callose in the plant cell walls.

Pathogen invasion first leads to local infection, and then local infection produces signals that are transported to the uninfected parts of the plant, making the uninfected part resistant to subsequent enlargement of the infection. This is called systemic acquired resistance (SAR) [90,91,92]. Signals generated by local infection include salicylic acid (SA), azelaic acid (AzA), glycerol-3-phosphate (G3P), and reactive nitrogen species (RNS) [93,94]. Lim et al. [95] studied the transport mechanism of SAR signals and found that PDLPs were involved. They treated plants with mock solution or avirulent strains, then detected SA, AzA, and G3P in apoplastic fluids and petiole exudates representing apoplastic and symplasmic communication signals, respectively. It was found that SA was transported through the ectoplasmic pathway, while G3P and AZA were transported through the symplastic pathway mediated through cell-wall PD conduits. Evaluation of SAR in *pdlp1/pdlp5* double mutant or *PDLP5*-overexpressing plants showed that PDLP1 and PDLP5 were involved in SAR. Moreover, PDLP1 can not only affect the transmission of SAR signals by regulating the permeability of PD, but also affect the stability of SAR signal transporter azelaic acid induced 1 (AZI1) through direct protein–protein interactions [96]. Ye et al. [97] also reported that a PDLP1 homolog, PDLP8, was able to interact with the acyl-CoA-binding protein 6 (ACBP6) to stabilize ACBP6 accumulation in PD.

Various hormone signaling pathways are associated with basal immune responses. The pathways activated by SA, a defense hormone, contribute to the responses [90]. When a pathogen infects plants, SA concentration is elevated. The expression of the PD regulator *PDLP5* was upregulated resulting in the restriction of cell-to-cell movement via PD [57]. Accumulation of PDLP5 protein in PD restricts PD permeability by stimulating callose deposition at the PD neck. Most notably, Cui and Lee [98] revealed that GSL6 (CALS1) is a primary partner for PDLP5. They produced a transcriptional reporter line *pGSL6*::GUS. GUS staining experiments confirmed that the *GSL6* expression profile is most highly correlated with that of *PDLP5* in SA-treatment experiments. In *gsl6-1* mutant plants, callose deposition did not change after treatment with SA. This indicates that SA-mediated callose production requires GSL6. The results of their experiments with carboxyfluorescein, a fluorescent dye that emits fluorescence and will not retrograde once it enters the cell, also support this conclusion. Neither changes in plasmodesmal callose deposition nor carboxyfluorescein movement were detected in *gsl6-1* mutant after SA treatment. Collectively, these sets of experimental evidence establish that GSL6 is a bona fide callose synthase gene regulating SA-dependent plasmodesmal responses. Therefore, PDLP5 requires GSL6 for SA-dependent plasmodesmal regulation. Evidence of subcellular localization of GSL6 to cell-wall PD is missing. Since the *gsl10* mutant plants are gametophytic lethal [99], the *gsl10* loss-of-function plants are unavailable. The roles of *GSL10* in regulating PD permeability and plant defense responses are still not investigated. Wang et al. [100] cloned and overexpressed the extracellular domain of *Arabidopsis* PDLP5 in an insect expression system, followed by purification, crystallization, and diffraction analysis. The extracellular domain of PDLP5 includes two DUF26 domains. They collected X-ray diffraction data to 1.90 Å resolution, which indicated that PDLP5 may function as a dimer. This research provides novel understanding of the molecular mechanisms behind PD permeability regulation and plant system immunity [100]. It is still far from clear, however, how PDLPs regulate callose synthase gene expression and callose deposition in PD [101].

### 4.5. Other PD-Related Cell Wall Proteins and Even More to Be Identified

The proteins and lipids of eukaryotes are sequentially organized in the plane of the membrane, forming microdomains that distinguish and coordinate biological processes, ensuring specific kinetics and functions to specific regions within the cell [102,103,104]. Grison et al. [105] used *Arabidopsis* cultured cells and primary roots to strictly isolate plasma membrane (PM) domains of PD. Immunogold labeling and Western blotting showed that many false-positive membrane proteins, such as PMA2, CesA3, CesA6, and PIP2, did not target to PD, but previously known PDLP1 and PDCB1 were specific to PD PM [105].

A hypothetical sterol carrier protein gene, *GHSCP2D*, was isolated from elongated cotton fibers by Zhang et al. [106]. They inhibited the expression of *GHSCP2D* and observed the deposition of callose on PD, confirming that *GHSCP2D* is involved in the permeability of PD. They demonstrated that the decrease of PD permeability was due to the inhibition of the expression of the PD-targeted beta-1,3-glucanase GhPDBG3 in cotton fibers. Both downregulation of *GHSCP2D* and the application of a sterol biosynthesis inhibitor can reduce the permeability of PD, indicating that sterol also has an effect on PD.

Viruses move through PD between plant cells. Park et al. [107] studied the role of PD in viral infection and found that NbEXPA1 is a PD-specific expansin, specifically involved in cell wall relaxation. They performed a label-free quantitative proteomic analysis of PD-enriched fractions of leaves infected with TuMV and healthy controls in *Nicotiana benthamiana*. They collected in total 1070 PD protein candidates. After data processing, 100 upregulated proteins and 48 downregulated proteins were identified in the TuMV-infected samples. The observation of YFP or GFP fused with NbEXPA1 showed that the fusion protein had a dotted pattern along the cell-wall boundary, suggesting that NbEXPA1 might be located in PD. Co-localization between NbEXPA1::YFP and PDLP1::CFP and between NbEXPA1::GFP and CI::RFP indicated that NbEXPA1 was indeed a PD-localized protein. The results of proteomics and RT-PCR analysis showed that TuMV infection downregulates the gene expression and protein accumulation of NbExpA1. Colocalization of NbEXPA1::YFP and TuMV-6K2::mCherry suggests that NbEXPA1 may be involved in the movement of the virus between cells. Infection of leaves with recombinant virus expressing CFP ligated with a viral RNA polymerase NIB (CFP::NIB), combined with a yeast two-hybrid assay and BiFC analysis, suggested that NbEXPA1 acts in the viral replication complex via interaction with NIB. Silencing and overexpression of *NbEXPA1* inhibits and promotes the movement of the virus between cells, respectively. The above results indicate that NbEXPA1 is a cell-wall protein associated with PD, which affects viral gene replication and its movement through the PD between cells through interaction with NIB.

## 5. Further Perspectives

**Figure 1:**
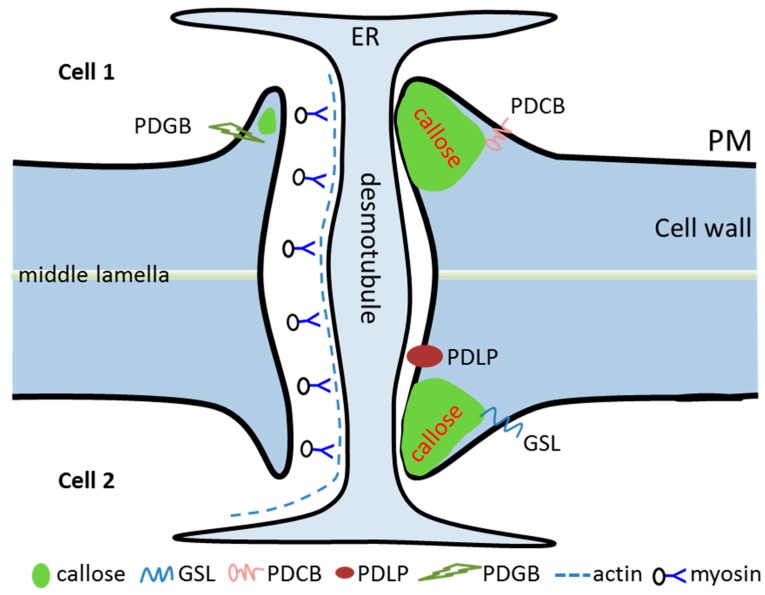
A simplified schematic representation of plasmodesmata (PD) structural and functional proteins. Permeability of PD in the plant cell wall is controlled by dynamic callose deposition (closed, right side) and degradation (open, left side) at the neck region of PD. ER, endoplasmic reticulum; GSL, glucan synthase-like; PM, plasma membrane; PDCB, PD-associated callose binding protein; PDGB, plasmodesmal associated β-1,3-glucanase; PDLP, PD-localized protein.

As the only channels for cytoplasmic communication between neighboring cells, PD are vital for the developmental process of plants. By combination of genetic, biochemical, and cell biology studies, many PD-associated proteins were identified and characterized (Figure 1). However, we still lack a fuller understanding of how the PD functions, which is largely due to the methodological difficulties in identification and characterization of PD components [108].

Increasing lines of evidence support that callose levels at the PD neck region play a central role in modulating the PD permeability and the symplasmic pathway. So far, the PD-related proteins, such as GSLs, PDGBs, PDCBs, and PDLPs, are all involved in maintaining callose homeostasis at the PD zone [28,29,36,53,56]. Further experiments are required to elucidate how transcription factors regulate the expression pattern of those genes in response to specific developmental cues and environmental stimuli. Since callose deposition and degradation in PD respond readily and rapidly to a wide range of signals, we speculate that PD-associated callose balancing proteins are mainly regulated at a post-transcriptional level. Another common feature is that those PD proteins contain transmembrane domains. Subcellular localization analysis showed that PD proteins are specifically targeted to PD embedded in the cell wall. It is also of interest to address the routes via which those proteins reach PD and more specifically the membrane of PD [109].

Previously, high-resolution electron microscopy and image processing methods greatly advanced our understanding of PD structure [110]. Immunochemical localization studies facilitated the detection of PD components. Indeed, our knowledge on PD is much dependent on technological progress. Despite their structural complexity, PD are important for plants and require further studies [21].

## Figures and Tables

**Table 1:** Proteins associated with plasmodesmata (PD).

Gene	Protein Function	Method	Biological Function	References
*Actin*	Cell cytoskeleton	Immunolocalization	PD structure component	[49,50,51]
*Myosin*	Cell cytoskeleton	Immunolocalization	PD structure component	[46,51,52]
*Tubulin*	Cell cytoskeleton	Immunolocalization	PD structure component	[51]
*GSL8* (*CALS10*)	Callose synthase	Genetics and cell biology	Callose deposition and auxin response	[53]
*GSL12* (*CALS3*)	Callose synthase	Genetics and cell biology	Callose deposition and phloem development	[36]
*PDBGs* (*AtBG_ppap*)	β-1,3-glucanase	Proteomics and cell biology	Callose degradation and lateral root development	[28,54,55]
*PDCBs*	Callose binding protein	Proteomics and cell biology	PD localization and callose stabilization at neck region	[29]
*PDLPs*	Transmembrane receptor-like protein kinase	Proteomics and cell biology	SAR- and SA-induced callose accumulation	[55,56,57,58,59]
